# Precision medicine in immune checkpoint blockade therapy for non-small cell lung cancer

**DOI:** 10.1186/s40169-017-0136-7

**Published:** 2017-01-20

**Authors:** Xiaoming Liu, William C. Cho

**Affiliations:** 1 Human Stem Cell Institute, General Hospital, Ningxia Medical University, Yinchuan, 750004 Ningxia China; 2 Department of Clinical Oncology, Queen Elizabeth Hospital, Kowloon, Hong Kong

**Keywords:** Immune checkpoint blockade, Non-small cell lung cancer (NSCLC), PD-1, PD-L1, Precision medicine, Nivolumab (Opdivo), Pembrolizumab (Keytruda), Atezolizumab (Tecentriq)

## Abstract

Immune checkpoint blockade therapy by targeting the programmed death protein 1/programmed death ligand 1 (PD-L1) axis using antibodies has yielded promising clinical responses in patients with non-small cell lung cancer (NSCLC). However, owing to the dynamic expression of PD-L1, degree of mutational/neoantigen load, intratumoral heterogeneity, infiltrated immune cells of tumor microenvironment of NSCLC, the response rates to these agents are limited, despite several companion diagnostic assays by detecting PD-L1 in tumor cells have been introduced into clinical practice. Therefore, in this era of precision medicine, there is an urgent need for predictive biomarkers to identify NSCLC patients likely to benefit from this novel therapy.

Lung cancer is a leading cause of cancer-related death worldwide, and non-small cell lung cancer (NSCLC) accounts for about 80–85% of all patients with lung cancer. Despite the pathogenesis of NSCLC are well researched and a number of novel drugs are being developed to target a variety of oncogenic drivers, such as epidermal growth factor receptor-tyrosine kinase inhibitors (EGFR-TKIs) (e.g. gefitinib, erlotinib, afatinib and osimertinib) and anaplastic lymphoma kinase (ALK) targeted agents, (e.g. crizotinib, alectinib, ceritinib and brigatinib), the presence of undruggable targets and the development of drug resistance limits the efficacy of treatment of targeted therapies for NSCLC. Encouragingly, the success of immune checkpoint blockade therapy in NSCLC has recently gained widespread recognition, with the scope to develop a diverse repertoire of synergistic and precise immunotherapeutics, although NSCLC was historically thought to be non-immunogenic [1].

An immune checkpoint blockade or inhibitor is designed to target inhibitory checkpoint molecules, such as programmed cell death protein 1 (PD-1), and its ligand programmed cell death protein ligand-1 (PD-L1) or cytotoxic T-lymphocyte-associated antigen 4 (CTLA-4) (Fig. 1) [2]. The promising therapeutic activity in both squamous and non-squamous carcinomas has led US Food and Drug Administration approval for two antibodies to PD-1, Nivolumab (Opdivo) (http://www.fda.gov/NewsEvents/Newsroom/PressAnnouncements/ucm466413.htm) and Pembrolizumab (Keytruda) (http://www.fda.gov/NewsEvents/Newsroom/PressAnnouncements/ucm465444.htm) [3], and one antibody to PD-L1, Atezolizumab (Tecentriq) (http://www.fda.gov/drugs/informationondrugs/approveddrugs/ucm525780.htm) [4] for NSCLC treatments with the prescribing information as an associated immunohistochemistry (IHC)-based companion or complementary diagnostic test for PD-L1 [5]. However, not all patients with advanced NSCLC benefit from these drugs. The response rates to these antibodies are only 15–20% in unselected NSCLC patients, suggesting there is a necessity to improve the response rate by selection of patients. The biomarkers for predicting the safety and efficacy of immune checkpoint blockade therapy currently remains lacking in NSCLC, which has significantly limited the harness of these potential agents as personalized medicine for the disease.

**Fig. 1:**
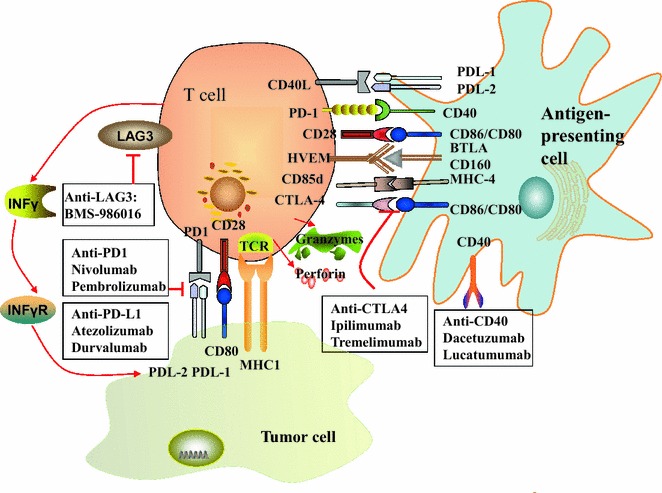
Schematic representation of immune co-stimulatory and co-inhibitory molecule receptors on T-cells and their ligands among T cells, antigen-presenting cells and tumor cells, and targeting strategies and agents of immune checkpoint blockade therapy. T-cell activation or inhibition induced by different co-stimulatory or co-inhibitory receptors (which are expressed on T-cells) bind to their ligands (which are expressed on dendritic cells or tumor cells). *BTLA* B- and T-lymphocyte attenuator, *CTLA*-*4* cytotoxic T-lymphocyte-associated protein 4, *HVEM* herpes virus entry mediator, *MHC* major histocompatibility complex, *PD*-*1* programmed cell death 1, *PD*-*L1* programmed cell death ligand-1

There are two different IHC assays approved for examining PD-L1 expression in formalin-fixed paraffin-embedded (FFPE) NSCLC tissue by FDA in October 2015, i.e. PD-L1 IHC 22C3 pharmDx linking to the use of pembrolizumab (Keytruda), and PD-L1 IHC 28-8 pharmDx linking to nivolumab (Opdivo) [3]. Recently, Roche also offered the VENTANA PD-L1 (SP142) Assay for atezolizumab (Tecentriq) in the assessment of PD-L1 protein in FFPE urothelial carcinoma and NSCLC tissues [6]. Indeed, results from the KEYNOTE-001 [7], CheckMate 057 [8], KEYNOTE-021 [9] and POPLAR [10] studies demonstrated that the efficacy of the PD-1/PD-L1 checkpoint blockades in advanced NSCLC patients was correlated with the expression of PD-L1. A better survival for pembrolizumab treatment was observed in patients with NSCLC who had more than 50% of PD-L1 positive tumor cells tested by PD-L1 IHC 22C3 pharmDx. With respect to nivolumab, no ideal biomarker has been identified yet, despite the IHC 28-8 pharmDx assay has been suggested as an accompanying diagnostic assay for the use of Opdivo. Indeed, a greater than 1% PD-L1-expressing tumor cells determined by this assay was associated with enhanced survival from nivolumab therapy versus docetaxel in non-squamous NSCLC patients [11]. However, it remains an open question whether nivolumab can be an alternative for those patients with fewer than 50% PD-L1 positive tumor cells tested by PD-L1 IHC 22C3 pharmDx, since nivolumab monotherapy showed an equivalent efficacy, not inferior to docetaxel for PD-L1-negative tumors, but superior to docetaxel for PD-L1-positive NSCLC, indicating that testing for PD-L1 in non-squamous NSCLC for decision of nivolumab regimen remains an option. These scenarios suggest that the predictability based on PD-L1 expression may differ between non-squamous NSCLC and squamous cell NSCLC [3]. With respect to the VENTANA PD-L1 (SP142) assay, the PD-L1 expression in ≥50% tumor cells or ≥10% immune cells in NSCLC tissue has been suggested to be associated with enhanced overall survival from atezolizumab (Tecentriq) [6]. Taken together, a precise selection of NSCLC patients who are most likely to benefit from immune checkpoint blockade therapies may maximize the benefit and reduce the high cost and unexpected immune-related adverse events (irAEs) of these therapies. Therefore, in the era of Precision Oncology, there is an unmet need for effective biomarkers that can predict the safety and efficacy of immune checkpoint blockade therapy [1, 11].

Despite PD-L1 expression has been used to identify good responders and long-term survivors in several clinical trials [12], multiple caveats are emerged in PD-1/PD-L1 based immune checkpoint blockade therapies when the sole PD-L1 expression is used as biomarker. Numerous factors, increase the technical difficulty of standardization for the interpretation of PD-L1 expression in clinical settings, including the assays, antibodies and cut-offs used, the usage of archival or fresh tissue, the heterogeneity of synchronous and metachronous tumor specimens. In addition to the PD-L1 expression in tumor cells, other genetic and epigenetic factors, such as tumor microenvironment/immune effector cells, non-synonymous mutation burden, and oncogene mutations in tumor cells, epithelial-to-mesenchymal phenotypes, and even smoking history were also found to be associated with objective response, durable clinical benefit, and progression-free survival (PFS) with immune checkpoint blockade therapy [11].

It has been recognized that PD-L1 is an inducible and dynamic biomarker subject to changes with tumor microenvironment (TME). Apart from being expressed in tumor cells, it is also expressed in tumor-infiltrating lymphocytes (TILs), in which PD-L1 expression may be more relevant to immune checkpoint blockade response than its expression in tumor cells, i.e. a PD-1 signaling inhibitor may have no effect on a PD-L1 expressing tumor lacking an appropriate immune infiltrate. In this regard, Smyth et al. [5] recently suggested a combination immunotherapy tailored to the TME based on the presence of PD-L1 expression and TILs. A TME phenotype with the presence of PD-L1 and TILs may benefit the most from PD1/PD-L1 blockade. Conversely, a TME phenotype with the absence of PD-L1 and lack of TILs may correlate with poor response to checkpoint blockades. For those NSCLC patients with a TME phenotype of PD-L1 constitutively expressed on tumor cells but lacking of TILs, or a phenotype of TME tumors containing TILs but absence of PD-L1, they will most unlikely be benefit from a PD-1/PD-L1 blockade therapy, other approaches or checkpoint blockades other than PD1/PD-L1 axis may be effective [5].

Beyond PD-L1 expression, an increasing interest has recently spurred in whether the mutational landscape affects responses to immune checkpoint blockade, owing to a high mutation rate is seen in NSCLC patients, which may strongly correlates with smoking history [13]. Furthermore, both EGFR mutation and ALK fusion can activate the PI3K/AKT signaling pathway, which in turn induced PD-L1 expression, suggesting a correlation of these mutations with anti-PD-1 antibody therapy. Indeed, patients with wild-type EGFR/KRAS mutations showed a greater benefit from nivolumab in terms of PFS and overall survival (OS) in the subgroup analysis of CheckMate057 study, while those with wild-type EGFR showed a better outcome in terms of OS from pembrolizumab compare to patients with EGFR mutations in the subgroup analysis of KEYNOTE-010 study [11]. In addition, NSCLC tumors with a higher non-synonymous mutation load (ultimately neoantigen burden) determined by exome sequencing, and low neoantigen intratumoral heterogeneity were correlated with significantly higher overall response rate (ORR) and durable clinical response to immune checkpoint blockade [11, 14]. However, such association is not absolute, some patients who carry a high mutation load also show non-response, a number of patients who harbor a low mutation load respond to the therapy [11]. This discordance may be attributed by the high degree of intratumoral heterogeneity (ITH) in NSCLC tumor, since sequencing using the bulk of tumor tissue may not fully capture the spatial complexity of the mutational landscape [14]. These findings clearly suggest that the response to immune checkpoint blockades is largely depended on the patients’ immune status and molecular subtypes. Together with the remarkable molecular diversity in NSCLC, these may support to a personalized approach to NSCLC immunotherapy based on the patients’ molecular characterization and immune status.

Unlike conventional and targeted cancer treatments, monitoring the responses of immune checkpoint blockades is a challenge in solid tumors, since there are no definitive radiologic criteria to differentiate between true progression and pseudo-progression available, although multiple nuclear probes serving to label and identify TILs are currently being tested in clinical trials [15], and the high-resolution PET imaging with therapeutic antibody-based PD-1/PD-L1 checkpoint tracers are also being tested in immunocompetent mice [16]. Intriguingly, by monitoring PD-L1 positive circulating tumor cells (CTCs) in nivolumab-treated NSCLC patients, Nicolazzo et al. [17] found that the patients with PD-L1(−) CTCs all obtained clinical benefit while those with PD-L1(+) CTCs all experienced progressive disease, suggesting a correlation of the persistence of PD-L1(+) CTCs and poor outcome of PD-1 blockade therapy.

With respect to precision medicine in immune checkpoint blockade therapy, the central goal is to tailor treatment to a patient’s individual immunological profile of both tumor and immune cells. In addition to test PD-L1, a multimodal approach including other immune inhibitory and stimulatory markers, TCR clonality, and somatic mutational burden may be more ideal predictors for patient identification and the response to therapy in NSCLC. Technically, the implementation of high-throughput sequencing analysis and genomic technologies are rapidly becoming part of standard practice, which may ultimately allow a more robust prediction of response to immune checkpoint blockade. For instance, if NSCLC patients who are identified with a high mutational load but with high degree of ITH and no clonal CD8 T cell infiltration by integrated approach, they may be represented a subtype that is unlikely to respond to immune checkpoint blockades [5]. Therefore, it is plausible to integrate both tumor and immune cell profiling with molecular data from the expression of checkpoint molecules and mutational burden to create the predictive assessments for patient selection, which may lead to an improved outcomes from immune checkpoint blockade therapy and pave the way for precision medicine to surmount NSCLC using these novel agents.
